# The Vulcanite Base

**Published:** 1859-02

**Authors:** 


### THE VULCANITE BASE.

We have received a great many inquiries in regard to this style of work. We can now say that the trade is open for the Profession.

Two rival companies, the "India Rubber Company," and the "Gutta Percha Company." The former propose to sell vulcanizing machines and rights for $50, with a perpetual tax of one dollar on each piece of work that may be made. The other sells machines and rights at $100, being free from any tax whatever.

We have recently had opportunities of seeing a considerable quantity of this work; and the more we see of it, the better we like it, as it is light and elastic, congeniel to the mouth, and apparently perfectly impervious to the secretions.

For more specific information of prices, manner of doing work, etc., see advertisements.

